# The impact of digital technology on entrepreneurship——Evidence from China General Social Survey

**DOI:** 10.1371/journal.pone.0310188

**Published:** 2024-09-24

**Authors:** Kedong Wu, Mengchun Zhu, Yahui Qu

**Affiliations:** 1 School of Economics and Management, Luoyang Institute of Science and Technology, Henan Universities and Colleges New Pattern Think Tank Industrial Innovation and Regional High Quality Development Research Institute, Luoyang, China; 2 School of Foreign Languages, Luoyang Institute of Science and Technology, Luoyang, China; Alexandru Ioan Cuza University: Universitatea Alexandru Ioan Cuza, ROMANIA

## Abstract

In light of the rapid development of digital technology, it is imperative to study the impact of digital technology on the labour force’s entrepreneurial choices with the utmost urgency. This paper first constructs a theoretical mechanism for how digital technology affects individual entrepreneurship. It then empirically examines data from the China General Social Survey (CGSS) to test the theory. The results show that digital technology significantly increases individual entrepreneurial choices. Furthermore, the conclusions of the study are robust even when the estimation method and variable measurement are changed. Finally, the study finds that digital technology has the greatest impact on entrepreneurship among individuals with low education, the second-largest impact on those with medium education, and the third-largest impact on those with high education. Individuals with higher education levels have the second largest impact on the entrepreneurship of individuals with higher education levels, while the smallest impact is observed in this group. Digital technology development has a stronger role in promoting entrepreneurship of individuals with urban household registration than those with rural household registration. In terms of sub-region, digital technology has a larger role in individual entrepreneurship in the eastern and central regions, and has a less significant role in the western region. The findings of this study suggest that there is a need to implement measures to accelerate the pace of digital technology development, enhance the training of entrepreneurial skills and attitudes among highly educated individuals, and direct efforts towards enhancing digital technology development in rural and western China.

## Introduction

In recent years, the development process of the digital economy in countries around the world has been accelerating. This has been accompanied by an acceleration in the updating and iteration of digital technology as a support for the development of the digital economy. Furthermore, digital technology has gradually penetrated into all areas of society, which has had a profound impact on the development of the national economy and the pattern of the labour market.

A number of scholars have studied the impact of digital technologies such as the Internet and artificial intelligence on the skill structure of the labour force. These include Berman, Falk, Bresnahan, Levy, Relich and Moore. Berman’s study found that the 1980s saw the emergence of new features in the structure of the US labour force as a result of technological advances [1]. The employment market is experiencing a notable surge in demand for skilled labour. Falk examines the relationship between information technology and labour force structure, concluding that an increase in information technology capital investment leads to an uptick in the demand for high-skilled labour, and that an increase in information inputs at the firm level will have a catalytic effect on high-skilled employment [2]. Bresnahan’s study indicates that digital technology has a significant skill bias, and the development of digital technology has resulted in a labour market that is biased towards skilled labour [3]. The studies of Levy, Relich, and Moore corroborate the conclusions of the aforementioned scholars and demonstrate that as the level of digital technology application increases, the labour market becomes biased towards a more highly educated, skilled, and qualified workforce [4–6].

The depth of research into the impact of digital technology on the industrial structure of the labour force has also attracted the attention of scholars.

Ding Lin and Wang Huijuan conducted an empirical examination of the relationship between Internet technology and employment, utilizing input-output data from several countries [7]. Their findings indicated that the advancement of Internet technology has a facilitating effect on overall employment, with a particularly pronounced impact on the employment of the labour force in the tertiary industry. Wang Wen conducted an empirical study on panel data for 30 provinces in China [8]. The empirical results indicate that industrial intelligence has the effect of reducing the share of employment in the manufacturing industry and increasing the share of employment in the service industry, with a particular increase in the share of employment in the productive service industry and high-end service industry. Guo Dongjie and colleagues demonstrated that the advancement of the digital economy is conducive to an increase in the share of employment in the tertiary industry and a decrease in the share of employment in the primary and secondary industries [9].

Concurrently, research on the impact of digital technology on labour force employment choice has begun to emerge. The existing literature on this topic primarily concerns the impact of digital technologies, such as the Internet, on the labour force’s employment choices. Overall, the use of the Internet for job searching can significantly reduce search costs and increase the likelihood of job seekers obtaining a job [10]. Furthermore, employers can release job information through online platforms to expand the scope of information transmission, thus reducing the time of job vacancies [11]. Additionally, job seekers can utilize Internet information resources to obtain relevant job information in a timely manner, which allows them to have more employment options and employment opportunities [12]. Furthermore, a number of scholars have examined the influence of the Internet on farm household entrepreneurship and family entrepreneurship [13, 14]. The study revealed that the utilization of the Internet fosters the growth of farm household and family entrepreneurship.

The current academic research on digital technology and labour force employment can be broadly categorized into three main areas: firstly, the relationship between digital technology and labour force employment skill structure; secondly, the relationship between digital technology and labour force employment industry structure; and thirdly, the relationship between digital technology and labour force employment choice. In comparison to the aforementioned two areas, research on digital technology and labour force employment choice is relatively scarce, with considerable scope for further investigation. The current findings primarily examine the influence of the Internet on labour force employment choice, with a paucity of studies that directly assess the impact of digital technology on labour force entrepreneurship. In the current era of the digital economy, the term "digital technology" encompasses not only the Internet but also a multitude of other content and a richer set of connotations. The mechanism by which digital technology affects individual labour force entrepreneurship remains unclear. The effect of digital technology on labour force entrepreneurship is still largely unknown. Furthermore, the impact of the development of digital technology on labour force entrepreneurship decision-making requires further investigation.

The potential contributions of this paper are as follows: Firstly, unlike previous studies, in that it this paper examines the phenomenon of labour force entrepreneurial choice from the perspective of digital technology development. This broadens the research perspective on the factors influencing labour force entrepreneurial choice. Secondly, this paper constructs a theoretical mechanism for the impact of digital technology on labour force entrepreneurship. This enriches the theoretical research content in this field. Thirdly, the theory will be empirically tested using micro-data, which will provide micro-empirical evidence for the theory of digital technology and entrepreneurship. Fourthly, the findings of this study will be incorporated into the policy recommendations for accelerating the development of digital technology in China. These recommendations will provide meaningful references for governmental decision-making.

## Theoretical analysis and research hypotheses

### Mechanisms by which digital technologies influence entrepreneurial decision-making

The development of digital technology can affect potential entrepreneurs’ entrepreneurial decisions through four paths: increasing entrepreneurial opportunities, improving the availability of information resources, expanding the scope of the market, and reducing the cost of entrepreneurship. Firstly, the impact of digital technology on entrepreneurial opportunities. Stevenson and Gumpert proposed that technology, market, government regulation and social values are the four external environmental factors affecting entrepreneurial opportunities [15]. Saemundsson and Dahlstrand based their classification of entrepreneurial opportunities on the two factors of technological knowledge and market knowledge [16], which they divided into four categories: existing technology—existing market, existing technology—new market, new technology—existing market and new technology—new market.—Expansion into new markets. The development of digital technology represents an important external environmental factor that will disrupt the established equilibrium, creating new entrepreneurial opportunities, production processes, markets, and ways of organizing. This will result in the emergence of two distinct types of entrepreneurial opportunities: the exploitation of existing markets through the application of new technology and the creation of new markets through the introduction of new technology. On one hand, the application of digital technologies to existing markets enables the introduction of new features to existing products and the enhancement of their performance. On another hand, significant innovations may be generated by the utilization of digital technology expertise to address novel requirements in both life and work contexts. Indeed, a considerable number of entrepreneurial opportunities can be generated in traditional industries or markets by leveraging digital technologies to meet consumer needs. Examples of this phenomenon include the application and popularization of e-commerce, online education, online healthcare and remote collaborative research and development.

Secondly, the impact of digital technology on access to information resources. Entrepreneurs require a variety of resources, including capital and information. The latter category encompasses a range of topics, including economic, policy, growth potential, market, technology, and other relevant areas. Shane and Venkataraman posit that information is instrumental in the utilization and development of entrepreneurial opportunities [17]. Consequently, digital technology can facilitate entrepreneurship by affecting access to information resources. On the one hand, the application of digital technology can assist potential entrepreneurs in more effectively identifying entrepreneurial information. Potential entrepreneurs who utilize digital technology are more likely to obtain a plethora of pertinent, readily accessible, punctual and efficacious information through digital technology, which enables them to identify and grasp potential entrepreneurial opportunities. On the other hand, digital technology can assist entrepreneurs in acquiring information regarding alterations in the entrepreneurial environment and in maintaining awareness of pertinent business-related counsel. This can facilitate timely, precise, and efficacious adjustments to entrepreneurial practices, thereby reducing the risk of the entrepreneurial process.

Thirdly, the impact of digital technologies on the scope of markets. The accelerated evolution of digital technologies has facilitated the efficient matching of producers and consumers, thereby reducing the costs associated with market research. The pervasive adoption of digital technologies has diminished the significance of geographical boundaries, enabling producers to connect with potential consumers at a greater distance than was previously feasible. This has facilitated entrepreneurs to reach a larger customer base at a reduced cost.

Indeed, digital technology, as a universal technology, has profoundly affected consumers’ food, clothing, housing and transportation, directly or indirectly impacting all industries. The pervasive adoption of digital technology has considerably broadened the market potential for aspiring entrepreneurs, enabling them to expand their market size at a significantly reduced cost. The expansion of the market due to the popularization and widespread use of digital technologies can increase the profitability of entrepreneurship. Furthermore, the expansion of the market can also increase the survival rate of a business, thereby reducing the risk of entrepreneurship.

Fourthly, the impact of digital technologies on the cost of entrepreneurship. The popularization and application of digital technology affect the cost of entrepreneurship in three principal ways. Firstly, the dissemination and utilization of digital technology can diminish the financial outlay required to commence a business. The advancement of digital technology facilitates the dissemination of information, thereby reducing the cost of acquiring it. The marginal cost of information provided by digital technology is minimal, and by utilizing digital technology, entrepreneurs can obtain all aspects of information they require for their own development at a reduced cost. Secondly, the extensive application of digital technology can reduce the variable cost of business participation in the market. The development of digital technology alters the structure of the market, reducing information asymmetry, thereby enhancing market efficiency. The dissemination of digital technology enables the expeditious completion of business transactions, thereby enhancing the efficiency of product activities. Finally, the utilization of digital technology can result in a reduction of the transaction costs associated with business operations. The advent of digital technology has brought with it the capacity for high-speed and easy data transmission, which has made it easier for entrepreneurs to exchange information with upstream and downstream firms. This has resulted in a notable reduction in transaction costs. The abundance, accessibility and transparency of information not only reduce search costs, but also supervision and enforcement costs.

In conclusion, we propose the following hypothesis:

Hypothesis 1: All other factors being equal, the development of digital technology can increase entrepreneurs’ options available to entrepreneurs.

### Heterogeneity in the impact of digital technology development on entrepreneurial decision-making

Firstly, digital technology may have a heterogeneous impact on the entrepreneurial choices of individuals with different levels of education. It is evident that educational attainment is a pivotal factor in understanding the influence of digital technology on entrepreneurial decision-making. Individuals with different levels of education demonstrate significant heterogeneity in their entrepreneurial motivations and opportunities when confronted with the advent of digitization. Those with higher levels of education are likely to possess more profound knowledge and professional skills, which affords them a significant advantage in the digital era. In the job market, they are more likely to find employment that aligns with their professional backgrounds and is compensated with relatively desirable salaries and benefits. Consequently, as digital technology continues to evolve, individuals with advanced academic qualifications may be more inclined to pursue secure career paths rather than embarking on entrepreneurial endeavors, despite the numerous advantages and opportunities that entrepreneurship offers. However, the situation is quite different for individuals with low levels of education. Those with lower levels of education are less competitive in the traditional job market and often face greater pressure to find employment and greater uncertainty regarding their future prospects. The advent of digital technology, particularly the emergence of mobile Internet and social media, has created a plethora of novel entrepreneurial opportunities for those with limited education. The advent of digital technology has facilitated the realization of entrepreneurial aspirations among individuals with low levels of education. These platforms have lowered the threshold for entrepreneurship, enabling individuals with low education levels to realize their self-worth through innovative business or service models. Those with a medium level of education occupy a position somewhere between the aforementioned extremes. While they may possess certain knowledge and skills, they also face certain employment pressures. Consequently, the advent of digital technology presents both opportunities and challenges for this demographic. Those with medium levels of education may choose between stable employment and entrepreneurship, according to their own circumstances and the prevailing market environment. In conclusion, the impact of digital technology on entrepreneurial choices is closely related to the level of education. The digital era presents individuals with different levels of education with distinct entrepreneurial opportunities and challenges. This reflects the universality and inclusiveness of digital technology. It is reasonable to posit that the development of digital technology has the most significant impact on the entrepreneurial choices of individuals with low levels of education, followed by those with medium levels of education, while the impact on individuals with higher levels of education is relatively minor.

Secondly, the impact of digital technology on entrepreneurial choices may vary depending on the domicile of the individual. In order to gain a more nuanced understanding of the impact of digital technologies on entrepreneurial choices, it is essential to consider the role of urban-rural household differences. These differences may lead to heterogeneous impacts across different household groups. Individuals with urban household registration are more likely to be situated in a more developed and diversified economic environment. In such an environment, individuals are more likely to be exposed to new technologies and new thinking, and are more likely to find resources and partners that are compatible with their entrepreneurial ideas. Conversely, towns and cities offer a larger market and stronger spending power, providing entrepreneurs with greater market opportunities. Furthermore, the infrastructure and public services in towns and cities are more comprehensive, and entrepreneurs can utilize more convenient channels and resources to support their business operations. These factors play a pivotal role in fostering entrepreneurial activities. Conversely, individuals with rural household registration may encounter greater challenges and constraints. Firstly, the relatively low level of economic development and limited market capacity in rural areas constrain the market opportunities for entrepreneurs. Secondly, the infrastructure and public services in rural areas are relatively underdeveloped, which makes it challenging for entrepreneurs to access the necessary resources. Furthermore, the dissemination of information in rural areas is relatively limited, which may impede entrepreneurs’ ability to obtain the most recent market intelligence and industry developments in a timely manner. This, in turn, increases the risk and uncertainty associated with entrepreneurship. Therefore, it is reasonable to assume that the development of digital technology has a greater contribution to the entrepreneurial choices of urban domiciled individuals. Nevertheless, this does not imply that individuals with rural household registration are unable to benefit from the advancement of digital technology. As technology becomes more prevalent and infrastructure improves, it is anticipated that entrepreneurs in rural areas will also be afforded greater opportunities and support.

Thirdly, the impact of digital technology on individual entrepreneurial choices may vary across different regions. In China, the impact of the development of digital technology on individual entrepreneurial choices is also significantly affected by geographical differences. Given China’s vast territory and the uneven economic and social development across its regions, there are significant differences in the level of development and the pace of digital technology adoption in the eastern, central and western regions. In the eastern and central regions, which have been at the vanguard of China’s economic expansion, the development of their digital economies has been particularly noteworthy. The digital technology sector in these two regions has a long history of development and has become deeply embedded in every aspect of society and the economy. Both large Internet companies and small stores at the end of the street are actively embracing digitization and seeking more efficient and convenient ways to operate. In such an environment, individual entrepreneurs are able to access the latest digital technologies and business models at an earlier stage and utilize them to enhance their competitiveness. Concurrently, the well-developed infrastructure and public services in these regions provide significant convenience for entrepreneurs. However, a different picture emerges when we turn our attention to the western region. Due to a number of historical and geographical factors, the development of digital technology in the western region has been relatively slow. Despite the state’s recent increase in investment in the western region, there persists a disparity between the western region and the eastern and central regions in terms of digital infrastructure construction, technology application and digital literacy. This discrepancy is also evident in the realm of individual entrepreneurship. In the western region, while digital technology offers entrepreneurs certain opportunities and conveniences, these are considerably less prevalent than in the eastern and central regions. Consequently, the development of digital technologies has had a relatively limited impact on individual entrepreneurial choices in the western region.

In conclusion, we put forth the following hypotheses:

Hypothesis 2a: There is a degree of heterogeneity in the choice of digital technology to influence entrepreneurship, all other input factors being equal.
Hypothesis 2b: Holding other input factors constant, there are differences between urban and rural areas in the choice of digital technologies to influence entrepreneurship.
Hypothesis 2c: It is assumed that there is regional heterogeneity in the choice of digital technology to influence entrepreneurship, provided that other input factors are equal.

## Research design

### Data sources and processing

The data presented in this paper has been derived from the following sources: The data used in this study was drawn from the CGSS 2013, 2015, 2017, 2018, and 2021 surveys, as well as the China Statistical Yearbook from previous years. The Chinese General Social Survey (CGSS) represents China’s earliest national, comprehensive, and continuous academic survey programme, implemented by the China Survey and Data Center of Renmin University of China. In accordance with international standards, more than 10,000 households are surveyed on each occasion in all provinces, municipalities and autonomous regions of mainland China. The most recent data from this survey is currently updated to 2021.

In this paper, we first merge the CGSS data for the five periods mentioned above. Second, we remove samples with missing key variables. Finally, it should be noted that the current regional matching code published by CGSS can only match provincial data. Therefore, we match micro data with provincial macro data to obtain the dataset used in this paper.

### Econometric modelling

Given that the explanatory variables in this paper are dummy variables, the Probit model is employed to assess the impact of digital technology development on individual entrepreneurship.:

$$entrepreneur_{it}=\alpha_{0}+\alpha_{1}index_{it}+\alpha_{2}X_{it}+Pro+Year+\varepsilon_{it}$$ (1)

There, *i* represents different provinces, and *t* represents different years, *entrepreneur* denotes the dummy variable for whether a labor force individual chooses to start a business or not, *index* denotes the level of digital technology development in the region where a labor force individual is located; *X* denotes the control variable for a labor force individual; in addition, *Pro* is used to denote the fixed effects of province, *Year* is used to denote the fixed effects of year, *ε* is used to denote the random error term.

### Variable selection

#### (1) The explained variable

Entrepreneur, is a dummy variable that measures whether or not an individual is engaged in entrepreneurial activity. If the individual is currently engaged in entrepreneurial activity, the variable entrepreneur is assigned a value of 1; otherwise, it is assigned a value of 0. The information on the current work status of the individual surveyed in the CGSS is used to determine whether or not the respondent is engaged in entrepreneurial activity. If the respondent indicates that they are a "boss" or "partner" during the interview, this is considered to be entrepreneurial activity. Additionally, "self-employed" and "freelance" are also considered to be entrepreneurial activities, as these are also existing activities. Furthermore, we consider self-employment and freelancing to be entrepreneurial activities, a common approach in existing literature.

#### (2) The core explanatory variables

Digital technology development level measurement index (index). At present, there is no unified standard for the index system to measure the level of digital technology development. In accordance with the methodologies employed in existing literature [18, 19], and in consideration of the availability of data, three dimensions are employed to capture the development of digital technology in each province in China: the construction of digital technology infrastructure, the scale of the digital economy, and the degree of digital technology mobile application. The length of long-distance fibre-optic cable lines is employed to assess the construction of digital technology infrastructure. The volume of express delivery business, software industry revenue, and total telecommunications business are utilized to gauge the scale of digital economy development. Finally, the number of end-of-year cell phone subscribers and the capacity of cell phone exchanges are employed to reflect the degree of mobile application of digital technology.

Firstly, the aforementioned six indicators were standardized utilizing the method of standardization of extreme deviation. To this end, it is necessary to ascertain the maximum value (Xmax) and minimum value (Xmin) of a specific indicator and calculate the extreme deviation. The ratio R is calculated as the difference between the maximum value (Xmax) and the minimum value (Xmin), and then the minimum value (Xmin) is subtracted from each observed value (X) of the variable in question. This value is then divided by the extreme deviation (R). Subsequently, the weight of each indicator is determined utilizing the entropy weighting method. Finally, the level of digital technology development is calculated based on the indicators and weights that have been standardized.

#### (3) Control variables

In this paper, control variables for individual characteristics and regional characteristics were selected.

Individual characteristics: The first variable is the gender of the respondent, which is assigned a value of 1 for males and 0 for females. The second variable is the age of the respondent, which is calculated as the square of the respondent’s age (age2). The third variable is whether the respondent has an urban household registration (huji), which is assigned a value of 1 for having an urban household registration and vice versa. Finally, the respondent’s membership of the Chinese Communist Party (CCP) was considered. This was categorized as either 1 for CCP membership or 0 for non-CCP membership. The marital status of the respondent is also taken into account. This is assigned a value of 1 for those who are married and 0 for those who are divorced. If the respondent is a member of the CPC (dangyuan), CPC members are assigned a value of 1, and non-CPC members are assigned a value of 0. The marital status of the respondent is determined by two indicators: whether or not the respondent has a spouse (spouse) and whether or not the respondent is divorced (divorce). If the respondent is married, the spouse is assigned a value of 1, and vice versa. The salary of the respondent is proxied by the logarithmic form of the respondent’s annual income.

Regional characteristics: The first indicator, population density (pop), is expressed as the ratio of the total population to the administrative area at the end of the year in the respondent’s province. The second indicator, employment rate (job), is expressed as the employment rate of urban units. The third indicator, financial development (loan), is measured as the average of the ratio of the total amount of loans from financial institutions to GDP. The fourth indicator is the openness level (tra), which is expressed as the ratio of the total amount of imports and exports in the respondent’s province to GDP. The fifth indicator is the urbanization rate (urban), which is expressed as a percentage. The sixth indicator is the regional economic development level (gdprio), which is measured as the real growth rate of regional GDP. This is expressed as the share of total imports and exports to GDP of the respondent’s province.

The descriptive statistics for the variables in this paper are presented in Table 1.

**Table 1:** Descriptive statistics of variables.

Variable	Obs	Mean	Std. Dev.	Min	Max
entrepreneur	37188	0.1011	0.3110	0.0000	1.0000
index	37188	0.1456	0.1448	0.0079	0.9150
gender	37188	0.4822	0.4997	0.0000	1.0000
age	37188	44.1135	12.9154	18.0000	65.0000
huji	37188	0.4439	0.4969	0.0000	1.0000
dangyuan	37188	0.0954	0.2937	0.0000	1.0000
salary	37188	10.2781	2.0850	4.3820	16.1181
spouse	37188	0.7871	0.4093	0.0000	1.0000
divorce	37188	0.0640	0.2448	0.0000	1.0000
pop	37188	3.3775	0.1911	2.7118	3.7650
job	37188	0.2329	0.0596	0.1539	0.4211
loan	37188	1.2714	0.4734	0.6553	2.5444
tra	37188	0.3348	0.3695	0.0321	1.5482
urban	37188	0.5814	0.1377	0.3496	0.8960
gdprio	37188	0.0949	0.0247	-0.0250	0.1640

## Analysis of measurement results

### Analysis of baseline regression results

Given that the explanatory variables in this paper are dummy variables, the Probit model is employed to assess the impact of digital technology development on individual entrepreneurship, while also accounting for province and year effects. In the robustness test section, the fixed effects model and Logit model are also employed for testing purposes. In order to examine the robustness of the model, the estimation is carried out by adding control variables step by step, and the regression results are shown in Table 2. It can be seen that in the estimation results (1)-(3), the marginal effects of the level of digital technology development on labour force entrepreneurship are all positive at the 1% significance level. This indicates that the development of digital technology significantly increases individual entrepreneurial choices, thereby confirming Hypothesis 1.

**Table 2:** Benchmark regression.

	(1)	(2)	(3)
	entrepreneur	entrepreneur	entrepreneur
index	0.3038***	0.2367***	0.5604***
	(0.0537)	(0.0596)	(0.0683)
gender		0.2469***	0.2471***
		(0.0181)	(0.0181)
age		0.1168***	0.1175***
		(0.0062)	(0.0062)
age2		-0.0016***	-0.0016***
		(0.0001)	(0.0001)
huji		-0.0072	0.0297
		(0.0185)	(0.0194)
dangyuan		-0.4039***	-0.4076***
		(0.0345)	(0.0346)
salary		0.0556***	0.0609***
		(0.0044)	(0.0044)
spouse		0.2830***	0.2679***
		(0.0323)	(0.0326)
divorce		0.1911***	0.1753***
		(0.0507)	(0.0508)
pop			0.0021
			(0.0490)
job			-0.5551**
			(0.2474)
loan			0.0939***
			(0.0342)
tra			-0.2662***
			(0.0511)
urban			0.0314
			(0.1349)
gdprio			2.2444***
			(0.4547)
_cons	-1.3095***	-4.0709***	-4.3378***
	(0.0116)	(0.1311)	(0.2405)
Province FE	YES	YES	YES
Year FE	YES	YES	YES
N	42663	37188	37188
Pseudo R^2^	0.0011	0.0668	0.0708

Standard errors in parentheses

* p < 0.10

** p < 0.05

*** p < 0.01.

### Heterogeneity discussion

#### (1) The existence of diverse levels of educational attainment

The sample was divided into three groups according to the education level of individuals: low (junior high school and below), middle (senior high school and junior college) and high (university college and above). The estimation results are shown in Table 3. The principal conclusions of this paper remain valid, and the improvement in the level of digital technology has increased the range of options available to individuals wishing to start their own business. However, the impact of digital technology on the entrepreneurship of individuals with different levels of education varies to some extent. The effect on individuals with low levels of education is the most pronounced, the effect on individuals with medium levels of education is the second most pronounced, and the effect on entrepreneurship of individuals with high levels of education is the least pronounced. The continuous development of digital technology has the effect of making it easier for highly educated individuals to find satisfactory jobs. Consequently, the level of digital technology development has a smaller effect on highly educated individuals relative to those with medium and low levels of education. Consequently, hypothesis 2a is validated.

**Table 3:** Estimates of heterogeneity in educational attainment.

	(1)	(2)	(3)
	Low educational attainment	Secondary education	High level of education
index	0.6189***	0.5019***	0.4013***
	(0.1014)	(0.1430)	(0.1331)
gender	0.1865***	0.2810***	0.3239***
	(0.0237)	(0.0384)	(0.0455)
age	0.0972***	0.1613***	0.1259***
	(0.0082)	(0.0132)	(0.0174)
age2	-0.0014***	-0.0021***	-0.0015***
	(0.0001)	(0.0002)	(0.0002)
huji	0.2787***	-0.1705***	-0.3244***
	(0.0275)	(0.0398)	(0.0624)
dangyuan	-0.1392**	-0.3555***	-0.4304***
	(0.0673)	(0.0664)	(0.0562)
salary	0.0659***	0.0554***	0.0615***
	(0.0056)	(0.0098)	(0.0124)
spouse	0.2486***	0.2735***	0.1053
	(0.0489)	(0.0652)	(0.0667)
divorce	0.1064	0.0880	0.2663**
	(0.0688)	(0.1045)	(0.1358)
pop	0.1463**	-0.1767*	-0.2197*
	(0.0649)	(0.0997)	(0.1237)
job	-0.6226*	-0.2750	-1.1171**
	(0.3505)	(0.5053)	(0.5409)
loan	0.1329***	0.0769	0.1594*
	(0.0446)	(0.0717)	(0.0895)
tra	-0.1113	-0.4126***	-0.3084***
	(0.0775)	(0.1007)	(0.1004)
urban	0.1323	-0.2740	0.2414
	(0.1882)	(0.2757)	(0.3070)
gdprio	2.3646***	0.9518	0.9297
	(0.5663)	(1.0264)	(1.2996)
_cons	-4.4783***	-3.8742***	-3.7628***
	(0.3243)	(0.4903)	(0.6110)
Province FE	YES	YES	YES
Year FE	YES	YES	YES
N	22241	7642	7305
Pseudo R^2^	0.0850	0.0985	0.0623

Standard errors in parentheses

* p < 0.10

** p < 0.05

*** p < 0.01.

#### (2) Urban-rural heterogeneity

The sample is divided into two groups, one comprising individuals residing in urban areas and the other in rural areas. Regression analysis was conducted on each group separately, and the results are presented in columns (1) and (2) of Table 4. The main conclusion of this paper remains valid. The development of digital technology increases the choice of individual entrepreneurship, but the impact of the development of digital technology on individual entrepreneurship of different domiciles shows some variability. This is evidenced by the greater positive promotion of entrepreneurship of individuals of urban domiciles, which confirms Hypothesis 2b. It is also notable that the coefficient of the impact of digital technology on entrepreneurship of individuals in towns and cities is significant at the 1% level, whereas the coefficient of the impact of digital technology on rural individual entrepreneurship is not significant. One potential explanation for this phenomenon is that individuals with urban household registration tend to have greater access to opportunities, larger markets, more convenient channels, and so forth, compared to rural individuals. Consequently, the development of digital technology has a more pronounced impact on the entrepreneurial choices of individuals with urban household registration.

**Table 4:** Estimates of urban-rural and east-central-west regional heterogeneity.

	(1)	(2)	(3)	(4)	(5)
	cities and towns	countryside	the east	central section	western part
index	0.3518***	-0.0594	0.4013***	2.1136**	-0.0641
	(0.1290)	(0.1447)	(0.1376)	(0.9869)	(0.4410)
gender	0.2574***	0.2383***	0.2889***	0.2301***	0.1922***
	(0.0273)	(0.0247)	(0.0285)	(0.0311)	(0.0374)
age	0.1454***	0.1057***	0.1541***	0.1006***	0.0899***
	(0.0097)	(0.0083)	(0.0102)	(0.0107)	(0.0120)
age2	-0.0018***	-0.0015***	-0.0020***	-0.0014***	-0.0013***
	(0.0001)	(0.0001)	(0.0001)	(0.0001)	(0.0001)
huji	0.0000	0.0000	-0.2216***	0.1615***	0.2504***
	(0.0000)	(0.0000)	(0.0310)	(0.0322)	(0.0400)
dangyuan	-0.5589***	-0.0635	-0.4076***	-0.3838***	-0.4470***
	(0.0430)	(0.0601)	(0.0498)	(0.0625)	(0.0780)
salary	0.0548***	0.0612***	0.0782***	0.0376***	0.0654***
	(0.0074)	(0.0058)	(0.0075)	(0.0073)	(0.0096)
spouse	0.1746***	0.3289***	0.1908***	0.3518***	0.2590***
	(0.0471)	(0.0459)	(0.0487)	(0.0624)	(0.0638)
divorce	0.0564	0.2332***	0.1275	0.1094	0.2460***
	(0.0704)	(0.0750)	(0.0787)	(0.0972)	(0.0949)
pop	-0.2257***	0.2406***	-0.1469*	0.3518**	0.5023**
	(0.0736)	(0.0673)	(0.0828)	(0.1634)	(0.2530)
job	-0.2672	-0.4204	0.4382	-4.0143***	0.9456
	(0.3968)	(0.4113)	(0.4057)	(0.7981)	(0.9673)
loan	-0.0222	0.1314**	-0.0883	-0.0904	0.2389***
	(0.0615)	(0.0518)	(0.0849)	(0.1910)	(0.0846)
tra	-0.1916**	0.1926*	-0.2186**	1.3993**	-0.1483
	(0.0894)	(0.1032)	(0.0906)	(0.6441)	(0.3395)
urban	-0.8000***	0.4436*	-0.2360	2.3211***	-0.3155
	(0.2016)	(0.2365)	(0.2898)	(0.5126)	(0.4082)
gdprio	0.2831	5.1370***	-1.2555	5.4457***	4.7687***
	(0.9371)	(0.7369)	(1.2991)	(1.4832)	(0.9606)
_cons	-3.3061***	-5.6361***	-4.0392***	-5.9804***	-6.3563***
	(0.3694)	(0.3370)	(0.4934)	(0.7584)	(0.9892)
Province FE	YES	YES	YES	YES	YES
Year FE	YES	YES	YES	YES	YES
N	17078	20110	15427	12537	9224
Pseudo R^2^	0.0867	0.0807	0.0932	0.0764	0.0731

Standard errors in parentheses

* p < 0.10

** p < 0.051

*** p < 0.01.

#### (3) Heterogeneity in the east, central and western regions

Further, based on the region where individuals are located, the sample is divided into three groups: East, Central and West. The estimation results are shown in columns (3)-(5) of Table 4. The main conclusion of this paper remains valid, namely that digital technology increases the choice of individual entrepreneurship. However, the impact of digital technology development on individual entrepreneurship in different regions shows a certain degree of variability. The effect on individual entrepreneurship in the central region is the largest and significant at the 5% level, that on the eastern region is the second largest and significant at the 1% level, and that on the western region is insignificant. This confirms Hypothesis 2c.

### Mechanical testing

The theoretical analysis in the previous section states that the development of digital technology can affect potential entrepreneurs’ entrepreneurial decisions through four paths: increasing entrepreneurial opportunities, improving the availability of information resources, expanding the scope of the market, and reducing the cost of entrepreneurship. This section uses a mediated effects model to test the theoretical mechanisms proposed in the previous section.

First, the entrepreneurial opportunity mechanism is tested. Considering that if the regional economy develops faster, then the entrepreneurial opportunities of the labour force may also be more, the economic development speed of the province where the labour force is located is used as a proxy variable for entrepreneurial opportunities and estimated based on the mediation effect model, and the results of the estimation are shown in columns (1)-(2) of Table 5. It can be seen that digital technology has a significant positive effect on entrepreneurial opportunities and that digital technology can increase the labour force’s options to choose entrepreneurship by generating entrepreneurial opportunities.

**Table 5:** Mechanical test.

	(1)	(2)	(3)	(4)	(5)	(6)	(7)	(8)
	gdprio	entrepreneur	market1	entrepreneur	market2	entrepreneur	market3	entrepreneur
index	0.0557***	0.0586***	2.3266***	0.0771***	11.3772***	0.0958***	6.7337***	0.0771***
	(0.0009)	(0.0181)	(0.0313)	(0.0143)	(0.0777)	(0.0168)	(0.0572)	(0.0156)
gender	-0.0002	0.0447***	0.0123	0.0446***	0.0437**	0.0447***	-0.0080	0.0448***
	(0.0002)	(0.0034)	(0.0079)	(0.0034)	(0.0196)	(0.0034)	(0.0144)	(0.0034)
age	-0.0002***	0.0156***	-0.0003	0.0156***	-0.0114*	0.0156***	-0.0067	0.0156***
	(0.0001)	(0.0010)	(0.0025)	(0.0010)	(0.0061)	(0.0010)	(0.0045)	(0.0010)
age2	0.0000***	-0.0002***	0.0000	-0.0002***	0.0002**	-0.0002***	0.0002***	-0.0002***
	(0.0000)	(0.0000)	(0.0000)	(0.0000)	(0.0001)	(0.0000)	(0.0000)	(0.0000)
huji	0.0021***	0.0044	0.0108	0.0037	0.0143	0.0039	-0.0749***	0.0043
	(0.0002)	(0.0037)	(0.0088)	(0.0037)	(0.0218)	(0.0037)	(0.0160)	(0.0037)
dangyuan	-0.0005*	-0.0651***	-0.0587***	-0.0648***	0.0495	-0.0658***	-0.0616**	-0.0654***
	(0.0003)	(0.0056)	(0.0132)	(0.0056)	(0.0327)	(0.0056)	(0.0240)	(0.0056)
salary	0.0003***	0.0109***	0.0020	0.0110***	-0.0528***	0.0111***	-0.0007	0.0111***
	(0.0000)	(0.0009)	(0.0020)	(0.0008)	(0.0049)	(0.0009)	(0.0036)	(0.0008)
spouse	-0.0005*	0.0580***	-0.0606***	0.0586***	0.2040***	0.0572***	0.0448*	0.0573***
	(0.0003)	(0.0059)	(0.0138)	(0.0059)	(0.0343)	(0.0059)	(0.0252)	(0.0059)
divorce	-0.0002	0.0460***	-0.0563***	0.0467***	0.0490	0.0457***	-0.0335	0.0459***
	(0.0004)	(0.0087)	(0.0205)	(0.0087)	(0.0509)	(0.0087)	(0.0375)	(0.0087)
pop	0.0047***	-0.0001	0.6424***	-0.0067	-5.4912***	0.0134	0.2015***	0.0027
	(0.0005)	(0.0093)	(0.0218)	(0.0094)	(0.0541)	(0.0105)	(0.0398)	(0.0093)
job	-0.0701***	-0.0706	-6.4965***	-0.0443	8.8427***	-0.1668***	-4.9884***	-0.1230***
	(0.0026)	(0.0506)	(0.1068)	(0.0477)	(0.2648)	(0.0462)	(0.1949)	(0.0459)
loan	-0.0085***	0.0052	-1.8706***	0.0500***	1.0067***	0.0174***	-1.3563***	0.0269***
	(0.0004)	(0.0074)	(0.0152)	(0.0077)	(0.0376)	(0.0065)	(0.0277)	(0.0067)
tra	-0.0185***	-0.0116	1.1483***	-0.0665***	-0.6909***	-0.0464***	0.0910**	-0.0481***
	(0.0006)	(0.0120)	(0.0217)	(0.0096)	(0.0539)	(0.0093)	(0.0396)	(0.0092)
urban	0.0270***	-0.0388	1.8602***	-0.0142	13.1234***	-0.0062	6.9205***	-0.0230
	(0.0014)	(0.0273)	(0.0578)	(0.0249)	(0.1434)	(0.0273)	(0.1055)	(0.0260)
gdprio		0.6284***	-3.2637***	0.3986***	9.0397***	0.3292***	5.4174***	0.3139***
		(0.1017)	(0.1947)	(0.0832)	(0.4829)	(0.0833)	(0.3553)	(0.0832)
market1				0.0165***				
				(0.0022)				
market2						0.0017*		
						(0.0009)		
market3								0.0057***
								(0.0012)
_cons	0.1172***	-0.3615***	8.1529***	-0.4882***	12.4849***	-0.3753***	4.2359***	-0.3778***
	(0.0022)	(0.0444)	(0.1033)	(0.0475)	(0.2561)	(0.0454)	(0.1885)	(0.0443)
Province FE	YES	YES	YES	YES	YES	YES	YES	YES
Year FE	YES	YES	YES	YES	YES	YES	YES	YES
N	37188	37188	37188	37188	37188	37188	37188	37188
R^2^	0.5663	0.0425	0.5526	0.0433	0.8015	0.0420	0.4629	0.0425

Standard errors in parentheses

* p < 0.10"

"** p < 0.05"

"*** p < 0.01

Secondly, the mechanism of information resource acquisition is tested. Considering that the higher degree of factor market development has a greater role in promoting the level of information technology development, the easier it is to access information resources, the degree of factor market development in the province where the labour force is located is used as a proxy variable for the ease or difficulty of access to information resources and estimated based on the mediated effects model, and the estimated results are shown in columns (3)-(4) of Table 5. It can be seen that digital technology has a significant positive impact on access to information resources, and digital technology can increase the labour force’s choice of entrepreneurship by enhancing access to information resources.

Thirdly, the market scope mechanism is tested. Considering that intermediary organisations arise and develop with the expansion of market scope, and that a higher degree of intermediary organisation development implies a greater market scope, the degree of intermediary organisation and legal development in the province where the labour force is located is used as a proxy variable for market scope and estimated based on the mediated effects model, and the results of the estimation are shown in columns (5)-(6) of Table 5. It can be seen that digital technology has a significant positive effect on market scope and that digital technology can increase the labour force’s options to choose entrepreneurship by expanding the market scope.

Finally, the entrepreneurial cost mechanism is tested. Considering that the government-market relationship is closely related to the institutional transaction costs of enterprise production and business activities, and that a good government-market relationship can reduce the transaction costs and financing constraints of enterprises, the government-market relationship is used as a proxy variable for entrepreneurial costs and estimated based on the mediated effects model, and the estimated results are shown in columns (7)-(8) of Table 5. It can be seen that digital technology has a significant positive impact on government-market relations, and that digital technology can increase the labour force’s choice to choose entrepreneurship by improving government-market relations and thus reducing entrepreneurial costs.

It should be noted that the three indicators used in this part of the discussion, namely the degree of development of factor markets, the degree of development of intermediary organisations and laws, and the relationship between the government and the market, are derived from the sub-indices in the China Marketisation Index compiled by Fan Gang.

### Robustness testing

#### (1) The instrumental variables approach

The regression results presented in Table 2 provide empirical support for the research hypothesis presented in the previous section. However, the estimation results are subject to endogeneity problems. To address the aforementioned issues, a re-estimation is conducted utilizing the instrumental variable method. The instrumental variable method requires the identification of exogenous variables that are related to digital technology and can only indirectly affect individual labour force entrepreneurship by influencing digital technology as instrumental variables of digital technology. In this paper, we utilize the distance from each provincial capital city to Hangzhou (in logarithmic form) as the instrumental variable for digital technology. The rationale is that digital finance, exemplified by Alipay, originated in Hangzhou, which has led to Hangzhou’s digital technology development being at the vanguard of the Chinese market. Based on the spatial spillover effect of digital technology, it is reasonable to assume that the closer the geographic proximity to Hangzhou, the higher the level of development of digital technology. There is currently no evidence to suggest that the distances of provincial capitals to Hangzhou can be used through channels other than those affecting the development of digital technology to affect labour force entrepreneurship. Furthermore, given that the distance from provincial capital cities to Hangzhou remains constant over time, the current study’s findings are employed to construct an instrumental variable that varies with region and time (index_iv1). This variable is derived from the mean value of digital technology development in other provinces in China. The first two columns of Table 6 present the results of the 2SLS estimation using instrumental variables. Among these, column (1) of Table 6 demonstrates the estimation of the first stage. It can be seen that the larger the value of the constructed instrumental variable, the lower the level of digital economic development of the region. The estimated coefficient is significant at the 1% level with an R2 of 0.6576, indicating that the instrumental variable has a strong explanatory power for the endogenous variables. Column (2) of Table 6 presents the estimation of the second stage. It can be observed that the coefficient of digital technology is significantly positive at the 1% level. This shows that the estimated results of the instrumental variables support the main hypothesis of this paper.

**Table 6:** Robustness test I: Instrumental variable approach.

	(1)	(2)	(3)	(4)
	index	entrepreneur	index	entrepreneur
index_iv1	-0.1389***			
	(0.0017)			
index_iv2			-0.0673***	
			(0.0034)	
index		1.3222***		1.3656***
		(0.2340)		(0.1534)
gender	-0.0005	0.2470***	-0.0011	0.2497***
	(0.0009)	(0.0182)	(0.0010)	(0.0182)
age	0.0001	0.1184***	-0.0000	0.1182***
	(0.0003)	(0.0063)	(0.0003)	(0.0063)
age2	-0.0000	-0.0016***	-0.0000	-0.0016***
	(0.0000)	(0.0001)	(0.0000)	(0.0001)
huji	-0.0004	0.0326*	0.0015	0.0358*
	(0.0010)	(0.0195)	(0.0011)	(0.0195)
dangyuan	0.0033**	-0.4071***	0.0046***	-0.4093***
	(0.0015)	(0.0348)	(0.0016)	(0.0347)
salary	0.0002	0.0593***	0.0006**	0.0579***
	(0.0002)	(0.0045)	(0.0002)	(0.0045)
spouse	0.0002	0.2657***	0.0022	0.2691***
	(0.0016)	(0.0327)	(0.0017)	(0.0326)
divorce	0.0022	0.1688***	0.0015	0.1751***
	(0.0023)	(0.0511)	(0.0025)	(0.0510)
pop	0.0399***	0.0035	-0.0238***	-0.0160
	(0.0025)	(0.0494)	(0.0027)	(0.0493)
job	0.8404***	-1.3680***	0.8332***	-1.0545***
	(0.0128)	(0.3674)	(0.0165)	(0.2631)
loan	-0.1692***	0.2085***	-0.1763***	0.1652***
	(0.0018)	(0.0539)	(0.0019)	(0.0363)
tra	0.3605***	-0.4577***	0.3921***	-0.3857***
	(0.0026)	(0.1024)	(0.0030)	(0.0554)
urban	-0.3451***	0.1752	-0.4503***	-0.0319
	(0.0070)	(0.1749)	(0.0079)	(0.1364)
gdprio	1.5829***	2.0081***	1.6896***	2.6765***
	(0.0255)	(0.6805)	(0.0278)	(0.4655)
_cons	-0.1182***	-4.3660***	0.0986***	-4.3203***
	(0.0117)	(0.2445)	(0.0143)	(0.2420)
Province FE	YES	YES	YES	YES
Year FE	YES	YES	YES	YES
N	37097	37097	37097	37097
Pseudo R^2^	0.6576		0.5978	

Standard errors in parentheses

* p < 0.10

** p < 0.05

*** p < 0.01.

Furthermore, given that Beijing, Guizhou, and Ulanqab are China’s national big data centres, regions that are closer to the big data centres are likely to have more favourable conditions for the development of digital technology. Therefore, this paper selects the average distance from the provincial capital to these three regions as the instrumental variable for estimation. Furthermore, given that the average distance remains constant over time, the findings of the current study are employed to construct an instrumental variable that varies with region and time (index_iv2). This variable is derived from the mean value of digital technology development in other provinces in China. The results of the regression are shown in the third and fourth columns of Table 6. Among these, column (3) of Table 6 demonstrates the estimation of the first stage. It can be seen that the larger the value of the constructed instrumental variable, the lower the level of digital economic development of the region. The estimated coefficient is significant at the 1% level with an R2 of 0.5978, indicating that the instrumental variable has a strong explanatory power for the endogenous variables. Column (4) of Table 6 presents the estimation of the second stage. It can be observed that the coefficient of digital technology is significantly positive at the 1% level. This again shows that the estimated results of the instrumental variables support the main hypothesis of this paper.

#### (2) Changing the estimation method and the measurement of the explanatory variables

In order to further test the robustness of the conclusions, this paper employs two alternative estimation methods for the model: the fixed effects model and the Logit model. The estimation results are presented in Table 7. Secondly, the measurement method of the explanatory variables is altered, with the measures of individual entrepreneurship utilizing "self-employment" (entrepreneur1) and "being his own boss" (entrepreneur2), respectively. The regression results are displayed in Table 8. Secondly, the explanatory variables were changed. The variables "self-employment" (entrepreneur1) and "own boss" (entrepreneur2) were used to measure individual entrepreneurship. The regression results are shown in Table 8. It can be seen that the conclusions of this paper maintain a good robustness.

**Table 7:** Robustness test II: Changing estimation methods.

	(1)	(2)	(3)	(4)
	entrepreneur	entrepreneur	entrepreneur	entrepreneur
index	0.0619***	0.0586***	0.5797***	0.4939***
	(0.0104)	(0.0181)	(0.1009)	(0.1734)
gender		0.0447***		0.4688***
		(0.0034)		(0.0343)
age		0.0156***		0.2344***
		(0.0010)		(0.0124)
age2		-0.0002***		-0.0031***
		(0.0000)		(0.0001)
huji		0.0044		0.0612*
		(0.0037)		(0.0367)
dangyuan		-0.0651***		-0.8093***
		(0.0056)		(0.0704)
salary		0.0109***		0.1086***
		(0.0009)		(0.0080)
spouse		0.0580***		0.5148***
		(0.0059)		(0.0635)
divorce		0.0460***		0.3266***
		(0.0087)		(0.0995)
pop		-0.0001		-0.0358
		(0.0093)		(0.0919)
job		-0.0706		-0.3042
		(0.0506)		(0.5228)
loan		0.0052		0.0480
		(0.0074)		(0.0733)
tra		-0.0116		-0.1405
		(0.0120)		(0.1246)
urban		-0.0388		-0.4643
		(0.0273)		(0.2847)
gdprio		0.6284***		7.4143***
		(0.1017)		(1.0828)
_cons	0.0921***	-0.3615***	-2.2505***	-8.2669***
	(0.0021)	(0.0444)	(0.0224)	(0.4644)
Province FE	YES	YES	YES	YES
Year FE	YES	YES	YES	YES
N	42663	37188	42663	37188
Pseudo R^2^	0.0008	0.0425	0.0011	0.0716

Standard errors in parentheses

* p < 0.10

** p < 0.05

*** p < 0.01.

**Table 8:** Robustness test III: Changing the measurement of explained variables.

	(1)	(2)	(3)	(4)
	entrepreneur1	entrepreneur1	Entrepreneur2	Entrepreneur2
index	0.0994*	0.3783***	0.7911***	0.7828***
	(0.0580)	(0.0730)	(0.0838)	(0.1078)
gender		0.1965***		0.3372***
		(0.0188)		(0.0354)
age		0.1084***		0.1006***
		(0.0065)		(0.0121)
age2		-0.0015***		-0.0013***
		(0.0001)		(0.0001)
huji		0.0075		0.1130***
		(0.0202)		(0.0364)
dangyuan		-0.4306***		-0.1533***
		(0.0375)		(0.0569)
salary		0.0433***		0.0947***
		(0.0047)		(0.0076)
spouse		0.2643***		0.1232**
		(0.0342)		(0.0591)
divorce		0.1732***		0.0741
		(0.0532)		(0.0945)
pop		-0.0290		0.1306
		(0.0509)		(0.0935)
job		-0.5113**		-0.3671
		(0.2600)		(0.4425)
loan		0.0472		0.1954***
		(0.0357)		(0.0636)
tra		-0.2033***		-0.3061***
		(0.0540)		(0.0881)
urban		-0.0391		0.2547
		(0.1410)		(0.2496)
gdprio		2.4754***		-0.0062
		(0.4734)		(0.8644)
_cons	-1.3671***	-3.8478***	-2.2884***	-6.0438***
	(0.0121)	(0.2504)	(0.0217)	(0.4565)
Province FE	YES	YES	YES	YES
Year FE	YES	YES	YES	YES
N	42663	37188	42663	37188
Pseudo R^2^	0.0001	0.0626	0.0116	0.0793

Standard errors in parentheses

* p < 0.10

** p < 0.05

*** p < 0.01.

#### (3) Estimation using sub-indicators of the core explanatory variables

In order to test the effect of sub-indicators of the level of digital technology development on individual entrepreneurship, the sub-indicators of the digital technology development index are brought into the model for estimation here respectively. Table 9 reports the regression results of the indicators of the volume of courier business (KD). The income of the software industry (SOFT), the total amount of telecommunication services (DX), the number of end-of-year subscribers of mobile telephones (YD), the capacity of mobile telephone exchanges (RL), and the long-distance fibre optic cable line length indicator (GL) were regressed. It can be observed that, with the exception of the long-distance fibre optic cable line length indicator, all the other indicators exert a significant positive influence on individual entrepreneurial decision-making. This supports the main conclusions of this paper to a certain extent.

**Table 9:** Robustness test IV: Estimation using sub-indicators of core explanatory variables.

	(1)	(2)	(3)	(4)	(5)	(6)
	entrepreneur	entrepreneur	entrepreneur	entrepreneur	entrepreneur	entrepreneur
KD	0.0480***					
	(0.0057)					
SOFT		0.0403***				
		(0.0069)				
DX			0.1117***			
			(0.0148)			
YD				0.1136***		
				(0.0183)		
RL					0.0929***	
					(0.0172)	
GL						-0.0066
						(0.0078)
gender	0.2462***	0.2456***	0.2467***	0.2461***	0.2458***	0.2455***
	(0.0181)	(0.0181)	(0.0181)	(0.0181)	(0.0181)	(0.0181)
age	0.1178***	0.1174***	0.1176***	0.1176***	0.1174***	0.1175***
	(0.0062)	(0.0062)	(0.0062)	(0.0062)	(0.0062)	(0.0062)
age2	-0.0016***	-0.0016***	-0.0016***	-0.0016***	-0.0016***	-0.0016***
	(0.0001)	(0.0001)	(0.0001)	(0.0001)	(0.0001)	(0.0001)
huji	0.0313	0.0326*	0.0336*	0.0329*	0.0310	0.0258
	(0.0194)	(0.0195)	(0.0194)	(0.0195)	(0.0194)	(0.0194)
dangyuan	-0.4059***	-0.4058***	-0.4053***	-0.4057***	-0.4053***	-0.4050***
	(0.0346)	(0.0346)	(0.0346)	(0.0346)	(0.0346)	(0.0346)
salary	0.0608***	0.0627***	0.0605***	0.0608***	0.0613***	0.0627***
	(0.0044)	(0.0044)	(0.0044)	(0.0044)	(0.0044)	(0.0044)
spouse	0.2678***	0.2686***	0.2680***	0.2667***	0.2667***	0.2666***
	(0.0326)	(0.0325)	(0.0325)	(0.0325)	(0.0325)	(0.0325)
divorce	0.1746***	0.1749***	0.1764***	0.1758***	0.1746***	0.1733***
	(0.0509)	(0.0508)	(0.0508)	(0.0508)	(0.0508)	(0.0508)
pop	0.0154	-0.0121	-0.0390	-0.0477	-0.0254	0.0202
	(0.0490)	(0.0493)	(0.0495)	(0.0501)	(0.0496)	(0.0490)
job	-0.7012***	-0.5768**	-0.3997	-0.4504*	-0.3214	-0.2800
	(0.2489)	(0.2498)	(0.2445)	(0.2455)	(0.2444)	(0.2447)
loan	0.1079***	0.1122***	0.1178***	0.1426***	0.1091***	0.0354
	(0.0345)	(0.0358)	(0.0351)	(0.0374)	(0.0359)	(0.0339)
tra	-0.2202***	-0.2161***	-0.2869***	-0.2987***	-0.2569***	-0.1451***
	(0.0494)	(0.0502)	(0.0522)	(0.0544)	(0.0526)	(0.0495)
urban	-0.0229	-0.2553*	0.1223	0.1868	0.1663	0.0381
	(0.1353)	(0.1454)	(0.1353)	(0.1361)	(0.1357)	(0.1393)
gdprio	2.4921***	1.9012***	2.9362***	2.4073***	2.2322***	1.8486***
	(0.4581)	(0.4505)	(0.4733)	(0.4597)	(0.4569)	(0.4513)
_cons	-4.7658***	-4.6767***	-5.0014***	-5.2076***	-5.1301***	-4.2614***
	(0.2459)	(0.2468)	(0.2558)	(0.2775)	(0.2807)	(0.2617)
Province FE	YES	YES	YES	YES	YES	YES
Year FE	YES	YES	YES	YES	YES	YES
N	37188	37188	37188	37188	37188	37188
Pseudo R2	0.0711	0.0696	0.0705	0.0698	0.0694	0.0684

Standard errors in parentheses

* p < 0.10

** p < 0.05

*** p < 0.01.

## Research findings and policy recommendations

### Conclusions of the study and discussion

This paper initially elucidates the mechanism by which the development of digital technology affects individual entrepreneurship theoretically and proposes research hypotheses. Secondly, it employs data from five periods of the China General Social Survey (CGSS) to test an econometric model and reaches the following conclusions: firstly, digital technology significantly increases individual entrepreneurial choices. Furthermore, the findings of the study remain robust after transforming estimation methods and changing the way variables are measured. Secondly, digital technology has the greatest impact on entrepreneurship among individuals with lower levels of education (i.e. those who have completed junior high school or below). It also has the second greatest impact on individuals with intermediate levels of education (i.e. those who have completed senior high school or secondary school). In contrast, it has the smallest impact on entrepreneurship among individuals with higher levels of education (i.e. those who have completed university college or above). Thirdly, the development of digital technology has a more pronounced effect on the promotion of entrepreneurship among individuals with urban household registration than among those with rural household registration. Fourth, regionally, the impact of digital technology on individual entrepreneurship is more pronounced in the East and Central regions.

In summary, this paper examines the phenomenon of labour entrepreneurship from the perspective of digital technology development. It broadens the scope of research on the factors influencing labour force entrepreneurship choices, enriches the theoretical content of digital technology and labour force entrepreneurship, and provides micro-empirical evidence for the theory of digital technology and entrepreneurship. The findings of the study will provide valuable references for government decision-making.

The findings of this paper are consistent with those of other current literatures. For example, studies have found that the Internet has a facilitating effect on entrepreneurial behaviour [10], that the construction of digital villages is conducive to entrepreneurship among rural residents [13], and that the use of robots and the development of digital finance are also conducive to entrepreneurship [20]. However, unlike these studies, which have all looked at one aspect of digital technology, the digital technology that is the subject of this paper is a larger category that expands and deepens these studies.

### Policy recommendations

The findings of this paper provide suggestions for promoting "mass entrepreneurship" in China from a digital technology perspective, with obvious policy implications. Firstly, it is recommended that the level of digital technology development be accelerated. It is recommended that a plan for the development of digital technology in China be formulated and implemented. This should include the strengthening of strategic guidance and policy support for the development of digital technology, as well as the improvement of laws and regulations on digital technology market access, operation, management, innovation, security, and so forth. This will create a favourable market environment, thereby providing institutional safeguards for the development of digital technology. Furthermore, the construction of digital China should be viewed as an opportunity to strengthen the construction of digital technology infrastructure, with the basic network system being given priority for the promotion of the development of digital technology. The following is a summary of the measures taken by the government to promote the development of digital technology.

Secondly, the cultivation of entrepreneurial practice and entrepreneurial spirit among highly educated individuals should be strengthened. The findings of this study indicate that digital technology has the least impact on the employment decision-making of the labour force with higher education levels. In other words, the higher the education level, the weaker the promotional effect of digital technology on individual entrepreneurship. Consequently, the entrepreneurial enthusiasm of college graduates can be stimulated by strengthening the innovation and entrepreneurial practice of college students, cultivating their entrepreneurial spirit as well as entrepreneurial thinking, and encouraging college students with entrepreneurial thinking and entrepreneurial ability to actively participate in the entrepreneurial army.

Thirdly, in order to achieve a balance, we focus on enhancing the development of digital technology in rural and western China. Empirical evidence indicates that digital technology has a stronger impact on the entrepreneurial decision-making of the labour force in the eastern and central regions and in towns and cities, while its impact on the entrepreneurial choices of the labour force in the western region and rural areas is relatively weaker. This suggests that the current low level of digital technology development in China’s western and rural regions may be hindering the entrepreneurial activities of individual laborers. Consequently, it is imperative to prioritize the balanced development of digital technology in China, accelerate the advancement of digital technology in the western region, and gradually narrow the disparity between urban and rural areas in terms of digitization, in accordance with the Chinese government’s "mass entrepreneurship" policy.

It is important to note that the research presented in this paper is based on data from China. However, the effects of digital technology on entrepreneurship can be reasonably generalised to other countries and regions. Consequently, the recommendations for countermeasures presented in this paper can also serve as a reference for policy formulation in countries other than China.

## Insufficient research and prospects

This paper examines the impact of digital technology development on labour entrepreneurship. However, the CGSS data only publishes codes that match provincial-level data, which limits the scope of the paper. This means that the paper is unable to find suitable exogenous shocks related to digital technology development to deal with the endogenous problems of this paper. This is a limitation of this paper.

Given these limitations, future research could consider utilizing data from other studies to determine the causal relationship between digital technology development and entrepreneurship with exogenous shocks to digital technology development. Furthermore, given that data elements are an important foundation for digital technology development, the relationship between data element development and entrepreneurship represents a promising direction for future research.

## Supporting information

S1 Data (XLSX)
